# Hereditary Breast and Ovarian Cancer Screening Syndrome Profile in Women Diagnosed with Breast Cancer from Paraná State Southwest

**DOI:** 10.1055/s-0041-1733998

**Published:** 2021-09-21

**Authors:** Juliana Batista de Moura, Carla Camila Ghedin, Érika Tomie Takakura, Thalita Basso Scandolara, Daniel Rech, Carolina Panis

**Affiliations:** 1 Laboratory of Tumor Biology, Universidade do Estado do Paraná, Francisco Beltrão, PR, Brazil; 2 Department of Genetics, Universidade Federal do Rio de Janeiro, Rio de Janeiro, RJ, Brazil; 3 Center of Health Sciences, Francisco Beltrão Cancer Hospital, Francisco Beltrão, PR, Brazil

**Keywords:** breast cancer, hereditary breast and ovary cancer syndrome, cancer screening, public health, câncer de mama, síndrome hereditária de câncer de mama e ovário, rastreamento de câncer, saúde pública

## Abstract

**Objective** This study evaluated the risk of the hereditary breast and ovarian cancer (HBOC) syndrome in patients with breast cancer by using the Family History Screening 7 (FHS-7) tool, a validated low-cost questionnaire with high sensitivity able to screen the HBOC risk in the population.

**Methods** Women diagnosed with breast cancer ( *n* = 101) assisted by the Unified Health System at the 8th Regional Health Municipal Office of the state of Paraná answered the FHS-7, and the results were analyzed using IBM SPSS Statistics for Windows, Version 25.0. software (IBM Corp., Armonk, NY, USA).

**Results** The risk of HBOC was 19.80% ( *n* = 20). Patients at risk exhibited aggressive tumor characteristics, such as high-grade tumors (30%), presence of angiolymphatic emboli (35%), and premenopausal at diagnosis (50%). Significant associations between the prevalence of high-grade tumors were observed in women younger than 50 years at diagnosis with HBOC ( *p* = 0.003).

**Conclusion** Our findings suggest a possible family inheritance associated with worse clinical features in women with breast cancer in this population, indicating that HBOC investigation can be initially performed with low-cost instruments such as FHS-7.

## Resumo

**Objetivo** Este estudo avaliou o risco da síndrome hereditária de câncer de mama e ovário (HBOC, na sigla em inglês) em pacientes com câncer de mama utilizando a ferramenta *Familial History Screening* 7 (FHS-7), um questionário validado de baixo custo e com alta sensibilidade capaz de rastrear o risco de HBOC na população.

**Métodos** Mulheres diagnosticadas com câncer de mama ( *n* = 101) assistidas pelo Sistema Único de Saúde da 8ª Regional de Saúde do estado do Paraná responderam ao questionário FHS-7, e os resultados foram analisados pelo software IBM SPSS *for Windows, Version* 25.0. (IBM Corp., Armonk, NY, EUA).

**Resultados** A ocorrência do risco de HBOC foi de 19,80% ( *n* = 20). Pacientes em risco exibiram características agressivas do tumor como tumores de alto grau (30%), presença de êmbolos angiolinfáticos (35%) e pré-menopausa ao diagnóstico (50%). Associações significantes foram observadas entre a prevalência de tumores de alto grau e diagnóstico abaixo de 50 anos no grupo HBOC ( *p* = 0.003).

**Conclusão** Nossos achados sugerem uma possível herança familiar associada a piores características clínicas em mulheres com câncer de mama nessa população, indicando que a investigação de HBOC pode ser realizada, inicialmente, com instrumentos de baixo custo, como o FHS-7.

## Introduction

Breast cancer is the major cause of women's death in the world. In Brazil, 60,000 new cases are estimated for 2020. 1 Around 5 to 10% of breast cancer cases occur due to inheritance of mutated genes in the DNA repair pathway, such as *BRCA1* , *BRCA2* , and *TP53* . 2 These mutations increase the probability of cancer in the breast, ovaries, ovarian tubes, peritoneum, prostate, intestine, and colon, among other neoplasms not well established in the literature yet. 3 Despite the high prevalence of breast cancer in the Brazilian population, there is a lack of studies conducted in patients with germline mutations and familial cancer. In addition, the southern region of Brazil presents the highest incidence of hereditary breast cancer, which usually results in tumors with poor prognosis and increased rates of death, social, and health costs, such as triple-negative tumors. 4 5

Age at diagnosis under 50 years old, pre-menopausal status, and being overweight at the time of diagnosis represent independent risk factors for the disease. 6 In comparison to luminal subtypes, that have estrogen and/or progesterone receptors, triple-negative (negative for all receptors) and human epidermal growth factor receptor 2 -positive (HER-2) breast cancers usually present high proliferation rates and are considered more aggressive tumors. 5 High histological grade, lymph node involvement, and presence of angiolymphatic emboli are also prognostic factors. 7 Young women (under 35 years old) with inherited breast cancer usually presents, worse disease characteristics, such as triple-negative tumors, which have few therapeutic options and low survival rates. 8 9

However, genetic testing for HBOC is not affordable for many patients, and the implementation of cancer counselling and testing in the public health system represents a challenge due to its high cost. A simple, low-cost, sensitive, and easily applicable instrument in primary care units would be particularly useful in underserved communities, where the identification and referral of high-risk individuals is difficult.

In this context, a Brazilian study conducted by Ashton-Prolla et al. 10 proposed the use of a screening instrument to assess the family history of breast cancer in individuals with an increased risk for hereditary syndromes, the Family History Screening 7 (FHS-7) questionnaire. This tool is composed by seven simple questions that are suggestive of HBOC, based on familial and patient cancer history. The instrument was validated for the Brazilian population attended in primary care unities, and consists in an adequate screening approach to identify at-risk individuals that could get benefit from genetic tests in the future.

As far as we know, the FHS-7 instrument has never been applied in the population of Southern Paraná. Thus, the present study aims to use the FHS-7 tool to screen breast cancer patients treated in a public oncology reference center to outline the syndrome profile in this population and investigate any clinicopathological correlation.

## Methods

This is an observational, descriptive, and prospective transversal study, approved by the ethics committee of the Universidade do Estado do Paraná (CAAE 35524814.4.0000.0107). Patients ( *n* = 350) attended in a public oncology hospital located in Francisco Beltrão, Paraná, which is the reference for 27 municipalities in the Southwest of Paraná under the 8th Regional Health Municipal Office of the State of Paraná and the Unified Health System, from January 2016 to August 2018. The patients were selected during routine doctor appointments, after analysis of image exams and/or biopsy results.

Eligible patients were women diagnosed with breast invasive ductal carcinoma (IDC) who volunteered and signed a consent form ( *n* = 101), and the inclusion criteria consisted in patients that were able to fully answer the questions, dichotomized as yes or no, in the FHS-7 questionnaire ( Chart 1 ), validated for the Brazilian population to evaluate individuals at HBOC risk. 10

**Chart 1:** Family History Screening 7 (FHS-7) questionnaire 10

Questions
1. Do you, or any first-degree relative, have or have had breast cancer?
2. Do you or any first-degree relative have or had bilateral breast cancer?
3. Has any man in your family had breast cancer?
4. Did any woman in your family have breast and ovarian cancer?
5. Has any woman in your family had breast cancer before the age of 50?
6. Do you have two or more relatives with breast and / or ovarian cancer?
7. Do you have two or more relatives with breast and / or bowel cancer?

Patients who responded positively to at least one of the questions were considered possible carriers of the syndrome. Based on the stratification of patients by the FHS-7 questionnaire, two groups were defined: the first was called HBOC-risk and was composed of those who answered positively to at least one of the questions in the questionnaire; and the second group was called sporadic cancer and was composed of patients who answered negatively to all questions.

The following parameters were collected from the patients' medical records: histological type of tumor (World Health Organization [WHO] classification); 11 estrogen, progesterone, and HER2 receptors status; molecular subtype (St. Gallen Consent); 12 tumor size; tumor grade (Nottingham prognostic index); 13 involvement of the surgical margins; presence of angiolymphatic emboli; age at diagnosis; body mass index (BMI), and menopausal status.

The variables were analyzed for statistical assumptions of normality (Shapiro-Wilk test) and homoscedasticity (Levene test). The results were assessed with the Grubbs test for the detection of outliers, and no outliers were detected. The frequency of the variables was determined as percentages, and the Person Chi-square and linear logistic regression tests were used to verify putative associations between the frequencies of the categorical variables (β and *p* -values were reported). For these analyses, the software IBM Statistical Package for the Social Science for Windows version 25.0 (IBM Corp., Armonk, NY, USA) was used. The statistical significance adopted for the analyses was 5%. Only the significant associations were reported in the study.

## Results

Among the patients, 80.2% answered negatively to all questions of the FHS-7 questionnaire, gathered in the “sporadic cancer” group ( *n* = 81). The remaining patients ( *n* = 20), the HBOC-risk group, answered positively to at least one of the instrument questions, which results in a prevalence of 19.8% for HBOC risk in the studied population. All patients included were Caucasian, agreeing with the characteristics of European colonization in the region studied.

The differences between the groups were not significant, and are only qualitatively described. There is a prevalence of negative hormone receptors (estrogen or progesterone) in the sporadic cancer group (46% and 52%, respectively), when compared with the HBOC-risk group (35% and 45%, respectively). Regarding the overexpression of HER2 receptors, there was a predominance in the HBOC-risk group in comparison to the sporadic cancer group (20% versus 12%, respectively). The clinicopathological characterization of patients is recorded in Table 1 .

**Table 1:** Clinicopathological characterization of the cohort

Parameters	Sporadic cancer	HBOC-risk	Chi-squared test
Molecular receptors status			
Absence of estrogen receptors	46% ( *n* = 37)	35% ( *n* = 7)	
Absence of progesterone receptors	52% ( *n* = 42)	45% ( *n* = 9)	*P* > 0.05
Absence of HER2 receptors	12% ( *n* = 10)	20% ( *n* = 4)	
Molecular subtypes Luminal A	36% ( *n* = 29)	45% ( *n* = 9)	*P* > 0.05
Luminal B	33% ( *n* = 27)	25% ( *n* = 5)	
Luminal-HER2/HER2	12% ( *n* = 10)	10% ( *n* = 2)	
Triple-negative	19% ( *n* = 15)	20% ( *n* = 4)	
Histological tumor grade			*P* > 0.05
Low	40% ( *n* = 32)	45% ( *n* = 9)	
Intermediate	42% ( *n* = 34)	25% ( *n* = 5)	
High	18% ( *n* = 15)	30% ( *n* = 6)	
Angiolymphatic emboli			*P* > 0.05
Presence	33% ( *n* = 27)	35% ( *n* = 7)	
Absence	67% ( *n* = 54)	65% ( *n* = 13)	
Age at diagnosis			*P* > 0.05
Under 50 years	40% ( *n* = 32)	35% ( *n* = 7)	
Above 50 years	60% ( *n* = 49)	65% ( *n* = 13)	
Body mass index (BMI, kg/m ^2^ )			*P* > 0.05
Eutrophic	36% ( *n* = 29)	50% ( *n* = 10)	
Overweight	40% ( *n* = 32)	25% ( *n* = 5)	
Obese	24% ( *n* = 19)	25% ( *n* = 5)	
Menopause Status at diagnosis			*P* > 0.05
Yes	52% ( *n* = 42)	50% ( *n* = 10)	
No	48% ( *n* = 39)	50% ( *n* = 10)	
Lymphnodal involvement			*P* > 0.05
Yes	24% ( *n* = 19)	25% ( *n* = 5)	
No	76% ( *n* = 62)	75% ( *n* = 15)	

Abbreviations: BMI, body mass index; HBOC, hereditary breast and ovarian cancer syndrome; HER2, human epidermal growth factor receptor-type2.

Both groups showed a similar distribution regarding molecular subtypes, age at diagnosis, menopause status and BMI classification as overweight/obese. The HBOC-risk group presented high-grade tumors more frequently than the sporadic cancer group (30% vs 18%, respectively), as well as intratumoral angiolymphatic emboli (35% vs 33% from the sporadic group) and lymph node involvement (25% vs 23% from the sporadic cancer group).

Regarding the HBOC-risk group, we found significant association between high histological grade and the occurrence of triple-negative tumors and high histological grade at diagnosis with age at diagnosis (under 50 years). Lymph node involvement was found to be significantly associated with the presence of angiolymphatic emboli in patients as well as being menopausal at diagnosis. The associations observed in the sporadic cancer group show a direct relationship between subtype and histological grade and between angiolymphatic emboli and lymph node involvement. The beta and *p* -values of β are described in Table 2 .

**Table 2:** Significant associations found in the HBOC-risk and sporadic cancer groups

**HBOC-risk**	**Beta value**	***p*** **-value**
Molecular subtype x histological grade	0.600	0.005
Age x histological grade	0.621	0.003
Angiolymphatic invasion x lymphnodal metastasis	0.545	0.013
Menopause at diagnosis x lymphnodal metastasis	0.577	0.008
**Sporadic cancer**	**Beta value**	***p*** **-value**
Molecular subtype x histological grade	0.225	0.045
Angiolymphatic invasion x lymphnodal metastasis	0.474	0.000

Abbreviation: HBOC, hereditary breast and ovarian cancer syndrome.

## Discussion

As far as we know, this is the first Brazilian study to apply the FHS-7 tool for screening the risk of HBOC in women with breast cancer in the Southwest region of Paraná, where most of its population is composed by European ancestry and inbreeding due to the geographic peculiarities of rugged relief. The prevalence of mutations in the *BRCA1/2* genes is associated with the occurrence of triple negative breast tumors and are higher in Caucasians when compared with Hispanics. 14 Furthermore, inbreeding has been identified as one of the possible causes for HBOC-associated mutations in *BRCA1/2* . 15 Thus, the studied population presents a favorable scenario for the occurrence of the HBOC syndrome.

The FHS-7instrument has high sensitivity for large-scale HBOC risk screening when compared with other molecular biology tools. 10 Thus, the analysis found that 16 patients (15.84%) have familial breast cancer diagnosed at early age, 5% have a first-degree family member with bilateral disease ( *n* = 5). No involvement was found concerning male relatives.

Regarding hormone receptors, patients in the HBOC-risk group had positive estrogen and progesterone receptors in only 35% and 45% of the cases, respectively, which may impair the response capacity to anti-hormone therapy and could impact the prognosis. In contrast, the sporadic cancer group showed an index of 46% for estrogen and 52% for progesterone. The normal functioning of the *BRCA1* gene protects breast tissue from genomic instability induced by estrogen, which could be associated with the better prognosis observed in tumors with positive hormonal receptors. 14

Increased high-grade tumors were found in the HBOC-risk group (45%), representing an important factor of worse prognosis and lower survival. 16 A similar result was found by Amendola & Vieira, according to whom patients with hereditary breast neoplasms had increased prevalence of high-grade disease, frequently related to negative estrogen and progesterone receptors. 17

In addition, the presence of intratumoral angiolymphatic emboli may predict the likelihood of lymph node invasion and could be a poor prognostic finding. 18 In our study, 35% patients of HBOC-risk group presented of angiolymphatic emboli, and 25% presented lymph node involvement. As for the body mass index (BMI), overweight and obesity are risk factors for development of postmenopausal breast cancer and may represent an additional risk for *BRCA1* and *BRCA2* mutation carriers, which are strongly associated with HBOC. 19 20 21 22 We found that 50% of the HBOC-risk group were classified as overweight (25%) or obese (25%).

Regarding age at diagnosis, older women had high-grade tumors. However, aging leads to the accumulation of free radicals, molecules capable of maintaining genomic instability that promotes cellular undifferentiating in breast tumors. 23 That is, both young and older patients have factors that contribute for the development of undifferentiated tumors. 24 In addition, the occurrence of high-grade tumors is also increased in *BRCA* -mutation carriers due to impaired DNA repair against lesions promoted by free radicals. 25

It is argued that even in the presence of lymph node involvement or tumors with aggressive subtypes, postmenopausal patients have a better prognosis when compared with those diagnosed younger than 35 years old. 24 The perspective of heredity among the patients in our study (19.8%), according to FHS-7, exceeds the average described by other national studies, such as the survey conducted by Dufloth et al., 26 which included Brazilian and Portuguese patients, and found a prevalence of familial breast cancer of 13%.

Exclusive associations were found in the HBOC group. Positive significant associations were identified concerning age at diagnosis and histological grade, indicating that older patients from the HBOC group have more undifferentiated tumors. The expression of BRCA1 is associated with familial breast tumors history; 14 15 its high expression has been reported in high grade cancers 27 and is believed to be a negative factor for patient survival. 28 However, no studies were found about the role of age in this context. Another significant relationship identified was the positive correlation between menopausal status at diagnosis and lymphnodal invasion in HBOC patients, suggesting that, in this group, the active hormonal status directly affects tumor invasiveness. These findings reinforce that the reproductive hormones have some additional impact that affects breast cancer aggressiveness in women at HBOC risk.

The limitations of the study include the small sample size, which precluded a more refined statistical design and the assessment of relevant outcomes, such as survival and relative risk. A strength of the study is that this kind of data are scarce in literature and add knowledge since they bring new information about the clinicopathological characteristics of women with breast cancer at HBOC risk in a geographical area where this syndrome seems to have a high prevalence.

## Conclusion

The present study used an investigation tool to evaluate the prevalence of HBOC risk in patients from the Southwest region of Paraná, whose prevalence exceeded the one found by other published studies in similar populations. Besides, patients at risk were associated with clinicopathological factors related with aggressive disease. The study indicates that the FHS-7 questionnaire could be used for the identification of patients at high risk for HBOC syndrome, enabling early detection and effective cancer prevention as well as genetic counselling.

## References

[1] Ministério da Saúde. Instituto Nacional de Câncer José Alencar Gomes da Silva Estimativa 2020: incidência de câncer no Brasil Rio de Janeiro INCA 2019

[2] Nielsen F C van Overeem Hansen T Sørensen C S Hereditary breast and ovarian cancer: new genes in confined pathways Nat Rev Cancer 2016 16 09 599 612 10.1038/nrc.2016.72 27515922

[3] Eleje G U Eke A C Ezebialu I U Ikechebelu J I Ugwu E O Okonkwo O O Risk-reducing bilateral salpingo-oophorectomy in women with BRCA1 or BRCA2 mutations Cochrane Database Syst Rev 2018 8 08 CD012464 10.1002/14651858.CD012464.pub2 30141832 PMC6513554

[4] Palmero E I Identificação e caracterização de pacientes em risco para câncer de mama hereditário no sul do Brasil [tese] Porto Alegre Universidade Federal do Rio Grande do Sul 2007

[5] Arpino G Milano M De Placido S Features of aggressive breast cancer Breast 2015 24 05 594 600 10.1016/j.breast.2015.06.001 26144637

[6] Schneider I J d'Orsi E Sobrevida em cinco anos e fatores prognósticos em mulheres com câncer de mama em Santa Catarina, Brasil Cad Saude Publica 2009 25 06 1285 1296 10.1590/S0102-311X2009000600011 19503959

[7] Curtit E Mansi L Maisonnette-Escot Y Sautière J L Pivot X Prognostic and predictive indicators in early-stage breast cancer and the role of genomic profiling: Focus on the Oncotype DX ^®^ Breast Recurrence Score Assay Eur J Surg Oncol 2017 43 05 921 930 10.1016/j.ejso.2016.11.016 28087099

[8] Reis-Filho J S Tutt A N Triple negative tumours: a critical review Histopathology 2008 52 01 108 118 10.1111/j.1365-2559.2007.02889.x 18171422

[9] Wang Y A Jian J W Hung C F Germline breast cancer susceptibility gene mutations and breast cancer outcomes BMC Cancer 2018 18 01 315 10.1186/s12885-018-4229-5 29566657 PMC5863855

[10] Ashton-Prolla P Giacomazzi J Schmidt A V Development and validation of a simple questionnaire for the identification of hereditary breast cancer in primary care BMC Cancer 2009 9 283 10.1186/1471-2407-9-283 19682358 PMC2739222

[11] Sinn H P Kreipe H A brief overview of the WHO Classification of Breast Tumors, 4th edition, focusing on issues and updates from the 3rd edition Breast Care (Basel) 2013 8 02 149 154 10.1159/000350774 24415964 PMC3683948

[12] Panel members Goldhirsch A Winer E P Coates A S Personalizing the treatment of women with early breast cancer: highlights of the St Gallen International Expert Consensus on the Primary Therapy of Early Breast Cancer 2013 Ann Oncol 2013 24 09 2206 2223 10.1093/annonc/mdt303 23917950 PMC3755334

[13] Galea M H Blamey R W Elston C E Ellis I O The Nottingham Prognostic Index in primary breast cancer Breast Cancer Res Treat 1992 22 03 207 219 10.1007/BF01840834 1391987

[14] Greenup R Buchanan A Lorizio W Prevalence of BRCA mutations among women with triple-negative breast cancer (TNBC) in a genetic counseling cohort Ann Surg Oncol 2013 20 10 3254 3258 10.1245/s10434-013-3205-1 23975317

[15] Loizzi V Cicinelli E Santamaria F BRCAmut and “founder effect”: a prospective study in a single academic institution Oncotarget 2018 9 32 22353 22358 10.18632/oncotarget.24959 29854283 PMC5976469

[16] Rakha E A Reis-Filho J S Baehner F Breast cancer prognostic classification in the molecular era: the role of histological grade Breast Cancer Res 2010 12 04 207 10.1186/bcr2607 20804570 PMC2949637

[17] Amendola L C Vieira R A contribuição dos genes BRCA na predisposição hereditária ao câncer de mama Rev Bras Cancerol 2005 51 04 325 330

[18] Song Y J Shin S H Cho J S Park M H Yoon J H Jegal Y J The role of lymphovascular invasion as a prognostic factor in patients with lymph node-positive operable invasive breast cancer J Breast Cancer 2011 14 03 198 203 10.4048/jbc.2011.14.3.198 22031801 PMC3200515

[19] Calle E E Kaaks R Overweight, obesity and cancer: epidemiological evidence and proposed mechanisms Nat Rev Cancer 2004 4 08 579 591 10.1038/nrc1408 15286738

[20] Kim J Y Lee D W Lee K H Prognostic role of body mass index is different according to menopausal status and tumor subtype in breast cancer patients Breast Cancer Res Treat 2019 176 02 453 460 10.1007/s10549-019-05249-1 31028608

[21] Inumaru L E Silveira E A Naves M M [Risk and protective factors for breast cancer: a systematic review] Cad Saude Publica 2011 27 07 1259 1270 10.1590/s0102-311X2011000700002Portuguese 21808811

[22] Guinan E M Hussey J McGarrigle S A A prospective investigation of predictive and modifiable risk factors for breast cancer in unaffected BRCA1 and BRCA2 gene carriers BMC Cancer 2013 13 138 10.1186/1471-2407-13-138 23517070 PMC3618255

[23] Benz C C Yau C Ageing, oxidative stress and cancer: paradigms in parallax Nat Rev Cancer 2008 8 11 875 879 10.1038/nrc2522 18948997 PMC2603471

[24] Freedman R A Partridge A H Management of breast cancer in very young women Breast 2013 22 02 S176 S179 10.1016/j.breast.2013.07.034 24074783

[25] Fridlich R Annamalai D Roy R Bernheim G Powell S N BRCA1 and BRCA2 protect against oxidative DNA damage converted into double-strand breaks during DNA replication DNA Repair (Amst) 2015 30 11 20 10.1016/j.dnarep.2015.03.002 25836596 PMC4442488

[26] Dufloth R M Carvalho S Heinrich J K Analysis of BRCA1 and BRCA2 mutations in Brazilian breast cancer patients with positive family history Sao Paulo Med J 2005 123 04 192 197 10.1590/s1516-31802005000400007 16389418 PMC11060407

[27] Hussein I A Ahmed S T Hameedi A D Immunohistochemical expression of BRCA1 protein, ER, PR and Her2/neu in breast cancer: a clinicopathological study Asian Pac J Cancer Prev 2020 21 04 1025 1029 10.31557/APJCP.2020.21.4.1025 32334465 PMC7445993

[28] Huszno J Kołosza Z Grzybowska E *BRCA1* mutation in breast cancer patients: Analysis of prognostic factors and survival Oncol Lett 2019 17 02 1986 1995 10.3892/ol.2018.9770 30675265 PMC6341769

